# Specific expression and function of the A-type cytochrome *c* oxidase under starvation conditions in *Pseudomonas aeruginosa*

**DOI:** 10.1371/journal.pone.0177957

**Published:** 2017-05-18

**Authors:** Tatsuya Osamura, Takuro Kawakami, Reiko Kido, Masaharu Ishii, Hiroyuki Arai

**Affiliations:** Department of Biotechnology, The University of Tokyo, Bunkyo-ku, Tokyo, Japan; East Carolina University Brody School of Medicine, UNITED STATES

## Abstract

*Pseudomonas aeruginosa* has one A-type (*caa*_3_) and multiple C-type (*cbb*_3_) cytochrome *c* oxidases as well as two quinol oxidases for aerobic respiration. The *caa*_3_ oxidase is highly efficient in creating a proton gradient across the cell membrane, but it is not expressed under normal growth conditions and its physiological role has not been investigated. In the present study, a mutant strain deficient in the *coxBA*-PA0107-*coxC* genes encoding *caa*_3_ exhibited normal growth under any test conditions, but it had low relative fitness under carbon starvation conditions, indicating that the expression of *caa*_3_ is advantageous under starvation conditions. A mutant that lacked four terminal oxidase gene clusters except for the *cox* genes was unable to grow aerobically because of low expression level of *caa*_3_. However, suppressor mutants that grew aerobically using *caa*_3_ as the only terminal oxidase emerged after aerobic subculturing. Analyses of the suppressor mutants revealed that a mutation of *roxS* encoding a sensor kinase of a two-component regulator RoxSR was necessary for the aerobic growth in synthetic medium. Two additional mutations in the 5′-flanking region of *coxB* were necessary for the aerobic growth in LB medium. Although the expression level of *caa*_3_ was higher in the suppressor mutants, their growth rates were lower than when the other terminal oxidases were utilized, suggesting that *caa*_3_ was not suited for utilization as the only terminal oxidase. Overexpression of the *cox* genes also inhibited the aerobic growth of the wild-type strain. These results indicate that *caa*_3_ is tightly regulated to be expressed only under starvation conditions at low level and it functions in cooperation with other terminal oxidases to facilitate survival in nutrient starvation conditions.

## Introduction

The bacterial A-type (*aa*_3_-type) cytochrome *c* oxidase is a member of the heme-copper oxidase superfamily and is closely related to the mitochondrial terminal oxidase [1]. This enzyme comprises of three core subunits and it catalyzes four electron reduction of oxygen to water at the end of the respiratory chain. The main catalytic subunit (subunit I) has a binuclear catalytic center, which comprises a high spin heme *a*_3_ and Cu_B_. Electrons are transferred from cytochrome *c* to the catalytic center via Cu_A_ and a low spin heme *a*, which are located in subunits II and I, respectively. The enzyme has two proton-conducting channels, the D-channel and K-channel [2, 3]. The *aa*_3_-type oxidases have a low affinity for oxygen and are usually expressed and functional under high oxygen conditions in many bacterial species, such as *Paracoccus denitrificans*, *Bradyrhizobium japonicum*, and *Rhodobacter sphaeroides* [4–7]. The homologous enzymes in *Thermus thermophilus* and *Bacillus subtilis* possess an additional heme *c* and are referred to as *caa*_3_-type [8, 9]. Cytochrome *c* is fused with subunit II in these enzymes.

The opportunistic human pathogen *Pseudomonas aeruginosa* was reported to have at least five terminal oxidases for aerobic respiration, including one A-type and two C-type (*cbb*_3_-1 and *cbb*_3_-2) cytochrome *c* oxidases, as well as A-type quinol oxidase (*bo*_3_) and cyanide insensitive oxidase (CIO), which is a non-heme-copper quinol oxidase. These oxidases are encoded by the *cox*, *cco1*, *cco2*, *cyo*, and *cio* gene clusters, respectively [10–12]. We have recently reported that *P*. *aeruginosa* has two additional gene clusters encoding the catalytic isosubunits of the C-type oxidase and multiple isoforms of the C-type oxidase could be produced via combinations of the multiple isosunbunits [13].

In the *cox* gene cluster (PA0105-0108), *coxB* (PA0105), *coxA* (PA0106), and *coxC* (*coIII*) (PA0108) encode subunits II, I, and III of the A-type cytochrome *c* oxidase, respectively. The product of the *cox* genes was reported to be the *aa*_3_ cytochrome *c* oxidase in previous reports [11–14]. However, CoxB has a heme *c* binding motif in its C-terminal domain, which is conserved in *caa*_3_ oxidases of *T*. *thermophilus* and *B*. *subtilis*, thus we refer to the enzyme encoded by the *cox* gene cluster as *caa*_3_ in the present study. PA0107 encodes a protein involved in the assembly of the enzyme complex. The genes located downstream of the *cox* genes, PA0112 and PA0113, encode enzymes that are probably involved in the production of heme *a* from heme *b* via heme *o*. PA0114 encodes a SenC/PrrC-type protein, which is involved in the delivery of copper to the heme-copper oxidases [15].

One of the high affinity C-type oxidases, *cbb*_3_-1, is constitutively expressed and has a major function in *P*. *aeruginosa*, even under high oxygen conditions. The *cox* genes are significantly induced only under nutrient starvation conditions and the expression levels of these genes are kept very low irrespective of oxygen tension under normal growth conditions [11, 14]. The *coxB* promoter depends on a stationary phase sigma factor, RpoS, and is repressed by a two-component regulator, RoxSR [11, 16]. RoxSR is a homolog of the RegBA/PrrBA-type regulators, which are thought to sense the electron flow of the electron transport chain (ETC) or the redox status of the quinone pool in purple non-sulfur photosynthetic bacteria [17, 18]. Recently, we reported that the ETC terminated by *caa*_3_ has the highest capacity to create a proton gradient across the membrane among the five terminal oxidases of *P*. *aeruginosa*, thereby indicating that *caa*_3_ is the most efficient at generating ATP per molecule of nutrient [14]. Therefore, it is reasonable to suggest that *caa*_3_ is utilized under starvation conditions in order to produce energy in an efficient manner. However, the physiological function of *caa*_3_ has not been investigated in *P*. *aeruginosa*.

In our previous study [14], we found that a quadruple mutant strain designated as QXAa, which was deficient in the four terminal oxidase gene clusters encoding two C-type cytochrome *c* oxidases and two quinol oxidases, was unable to grow under aerobic conditions, thereby suggesting that the *cox* genes were tightly repressed or the gene product was not functional in the mutant. However, suppressor mutants emerged after aerobic incubation, which could grow aerobically. In the present study, we investigated the fitness effect of *caa*_3_ under starvation conditions using a *cox* gene knockout mutant. We also investigated the regulatory mechanism of the *cox* genes based on analyses of the suppressor mutations.

## Materials and methods

### Bacterial strains and growth conditions

The bacterial strains and plasmids used in this study are described in S1 Table. *P*. *aeruginosa* PAO1ut, which is the PAO1 strain subcultured in our laboratory [11], was used as the wild-type stain (WT). *Escherichia coli* JM109 [19] was used as a host for the construction of plasmids. *E*. *coli* S17-1 [20] was used for the conjugative transfer of plasmids to *P*. *aeruginosa*. Cells were grown routinely in LB medium at 37°C. Synthetic medium [11] supplemented with 8 or 0.6 g/l glucose or 8 g/l sodium glutamate (glutamate medium) was used as necessary. The media were supplemented with 100 mM sodium nitrate for anaerobic cultivation. Erlenmeyer flasks or test tubes with gas-permeable plugs were used for aerobic cultivation. Test tubes sealed with butyl rubber stoppers were used for anaerobic cultivation and the gas phase was replaced with argon. Cell growth was monitored by measuring the optical density of the culture at 600 nm using a mini photo 518R (TAITEC, Saitama, Japan) for the test tube cultures and a JASCO V-630 BIO spectrophotometer (JASCO, Tokyo, Japan) for the cultures in Erlenmeyer flasks. The concentrations of the antibiotics were as follows: 100 μg/ml ampicillin (Ap) and 12.5 μg/ml tetracycline (Tc) for *E*. *coli*; and 200 μg/ml carbenicillin (Cb), 150 μg/ml Tc, 300 μg/ml streptomycin (Sm), and 30 μg/ml gentamicin (Gm) for *P*. *aeruginosa*. When necessary, 5% (w/v) sucrose or 0.5 mM IPTG was added to the medium.

### Construction of mutant strains

The recombinant DNA methods have been described previously [14]. A single mutant strain SAa, which lacked the *coxBA*-PA0107-*coxC* gene cluster, was constructed by in-frame deletion using the plasmid pEX-Δcox, as described previously [14].

PAO1ut-Sm and SAa-Tc were constructed by inserting the Sm and Tc resistance genes into the chromosomes of PAO1ut and SAa, respectively, using a Tn*7*-based method as described previously [11, 21]. mini-Tn*7*-Sm and mini-Tn*7*-Tc, which are derivatives of pUC18T-mini-Tn*7*T-Gm [21], were used to move the Sm and Tc resistance genes, respectively. mini-Tn*7*-Sm was constructed by inserting a 2.0-kb HindIII fragment carrying the Sm resistance gene from pHP45Ω [22] into the HindIII site of pUC18T-mini-Tn*7*T-Gm. To construct mini-Tn*7*-Tc, the Tc resistance gene was amplified from pBR322 with the primers pBR-f and pBR-r. After digesting with SacI and KpnI, the gene fragment was inserted into the appropriate site in pUC18T-mini-Tn*7*T-Gm. The primers used in this study are listed in S2 Table.

QXAacoxLac, QXAacioLac, QXAaS1coxLac, and QXAaS1cioLac, which carried the *lacZ* fusions on the genomic DNA and were used to the promoter assay of the *cox* or *cio* genes, were constructed by using pUC-cox-lacZ or pUC-cio-lacZ by the same procedure for construction of PAcoxLac and PAcioLac [11].

### Sequence analysis

The genome of QXAaS2 was sequenced using a Genome Analyzer II (Illumina, San Diego, CA) as a custom service from Takara Bio (Otsu, Japan) and compared with the genome sequence of PAO1ut which was sequenced using a MiSeq (Illumina). The sequence of the *coxBA*-PA0107-*coxC* genes and its 5′-flanking region, as well as the *roxSR* genes from QXAa, QXAaS1, and QXAaS3 were sequenced with a 3130*xl* Genetic Analyzer (ABI, Foster City, CA) after PCR amplification.

### 5′-RACE

The transcription start point of the *coxB* promoter was determined by the rapid amplification of C terminal end (5′-RACE) method using the 5′-Full RACE Core Set (Takara Bio). Total RNA was isolated from the PAO1ut cells at the exponential phase in glutamate medium, as described previously [11]. Single strand cDNA was synthesized from the RNA with the coxB-P primer. cDNA was circularized and/or concatemerized by T4 RNA ligase and then used as a template for the first round of PCR with the primers coxB-A1 and coxB-S1. The amplified fragments were used as templates for the nested PCR with the primers coxB-A2 and coxB-S2. The amplified fragments were inserted into the pGEM-T Easy Vector (Promega, Madison, WI) and sequenced using an ABI 3130*xl* Genetic Analyzer.

### Construction of plasmids and enzyme assays

The *lacZ* fusion plasmids used to measure the promoter activity of *coxB* were derivatives of the *lacZ* promoter probe vector pQF50 [23]. Schematic representation of the method used for construction of the fusion plasmid is shown in S1 Fig. Schematic diagram of the promoter and regulatory regions of the constructed fusion plamids is shown in S2 Fig. To construct the transcriptional fusion plasmids, the *coxB* promoter region was amplified by PCR with the primer sets coxZ1/coxZ2, digested with PstI and HindIII, and inserted into the appropriate sites in pQF50 (S1 Fig). The *coxB* promoter fragments, with no mutation or with two mutations at the SBS (insertion of A or C at position –151 from the initiation codon of *coxB*) and at the RBS (A to G point mutation at position –18 or G to A point mutation at position –9 from the initiation codon of *coxB*), were amplified using the chromosomal DNA of the WT or the suppressor mutants, respectively, as templates. To construct the *coxB* promoter fragment with a mutation only at SBS, the SBS fragment and the RBS fragments were amplified with the primer sets coxZ1/coxZ4 and coxZ2/coxZ5 using the chromosomal DNA from the suppressor mutant and WT, respectively. The entire promoter region was amplified with the primer set coxZ1/coxZ2 using the mixture of amplified SBS and RBS fragments as templates. The promoter fragment, with a mutation only at RBS (A to G point mutation at position –18 from the initiation codon of *coxB*), was created in the same manner using the chromosomal DNA from the WT and the suppressor mutants to amplify the SBS and RBS fragments, respectively.

The translational fusion plasmids were constructed by in-frame fusion of the initial three triplets of *coxB* with the reading frame of *lacZ* on the transcriptional fusion plasmids (S1 Fig). The primers coxZ7 and coxZ8 were designed to fuse the reading frames of *coxB* and *lacZ*. The PCR fragments containing the promoter region with the N-terminus of *coxB* and the *lacZ* gene were amplified from the transcriptional fusion plasmids with the primer sets coxZ1/coxZ8 and coxZ7/coxZ9, respectively. The mixture of two fragments was used as a template to amplify the fragments carrying the *coxB*::*lacZ* translational fusion with the primer set coxZ1/coxZ9. The amplified fragment was digested with PstI and replaced with the PstI fragment of pQF50, thereby obtaining the translational fusion plasmid. The primers coxZ7-S3 and coxZ8-S3, which comprised the sequence of QXAaS3, were used instead of coxZ7 and coxZ8 to construct the translational fusion plasmid with a point mutation at RBS. The β-galactosidase activity was determined according to a standard protocol [19].

pMMB-cox was used to overexpress of the *cox* genes in *P*. *aeruginosa* strains. The plasmid was constructed by inserting a 4.2-kb PCR fragment carrying the *coxBA*-PA0107-*coxC* genes, which was amplified from PAO1ut chromosomal DNA with the primers PAcox_F1 and PAcox_R1, into the EcoRI-HindIII sites of pMMB67EH [24]. Cytochrome *c* oxidase activity was visualized using the Nadi assay according to the method described previously [13]. Cells of overnight cultures were suspended in 1 ml of 20 mM Tris-HCl (pH 7.5). After adding 200 μl of a 1:1 mixture of 35 mM α-naphthol in ethanol and 30 mM *N*,*N*-dimethyl-*p*-phenylenediamine monohydrochloride to the cell suspension, the development of blue color by the formation of indophenol blue was observed. The reaction mixtures were incubated for 5 min at room temperature.

### Spectral analysis

Membrane fractions of the cells of PAO1ut, QXAaS1, and QXAaS2 were prepared according to a previously described method [14] and diluted to a protein concentration of 2 mg/ml using 33 mM potassium phosphate buffer (pH 7.0). Next, 1 ml aliquots of the membrane fraction were reduced with 4 mg sodium dithionite or oxidized with 0.5 mg ammonium persulfate. The differences in the reduced minus oxidized spectra were recorded at room temperature using a JASCO V-630 BIO spectrophotometer. The content of heme *a* was estimated from the spectra using a molar extinction coefficient of 11.6 mM^-1^cm^-1^ [25].

### Measurement of intracellular reactive oxygen species (ROS)

Endogenous levels of ROS were assayed with 2′,7′-dichlorodihydrofluorescin diacetate (DCFH-DA) staining by using OxiSelect^™^ Intracellular ROS Assay Kit (Cell Biolabs Inc., San Diego, CA). The bacterial strains were cultivated aerobically in LB medium supplemented with 0.5 mM IPTG. After cultivation for 3 h, a 1 mL aliquot of the culture was mixed with 1 ml of 20 μM DCFH-DA. After incubation for 1 h at 37°C, cells were collected, washed three times with PBS, and resuspended in PBS. The DAF fluorescence intensities (excitation and emission of 488 and 540 nm, respectively) of cells were measured with a JASCO FP-8200 spectrofluorometer and normalized by dividing by optical density at 600 nm of the cultures.

### Competitive index and relative fitness

Competitive index (CI) and relative fitness (w) represent the total effect of a particular genotype on the capacity for survival and reproduction under a particular condition. CI is commonly used to estimate the fitness cost of a mutation. CI was calculated by the following equation [26]: CI = (x1/y1)/(x0/y0), where x0 and x1 are the initial and final Colony-forming units (CFU) ml^−1^ for the mutant, respectively, and y0 and y1 are the initial and final CFU ml^−1^ for the WT, respectively. Because the CI value differs according to the growth phase [26], we also compared the w values. The w value of the mutant strain was calculated by the ratio of Malthusian parameters, which is essentially the ratio of the number of doublings, according to the following equation [27]: w = ln(x1/x0)/ln(y1/y0).

For determination of the CI and w values, equal amounts of precultures of strains PAO1ut-Sm and SAa-Tc, which were grown aerobically in LB medium, were mixed and cultivated in synthetic medium supplemented with sufficient (8 g/l) or restricted (0.6 g/l) amounts of glucose. A series of dilution of the mixed cultures immediately after inoculating the cells and after aerobic cultivation for 11 h were plated on LB plates containing Sm or Tc and incubated aerobically overnight at 37°C. CFUs of PAO1ut-Sm and SAa-Tc were determined by counting the colonies on the plates containing Sm and Tc, respectively.

### Statistical analysis

The Student’s *t* test was performed to evaluate significant differences in the experiments. Analysis was done by Microsoft Excel (Microsoft, WA).

## Results and discussion

### Effect of *caa*_3_ on the relative fitness under starvation conditions

**Fig 1:**
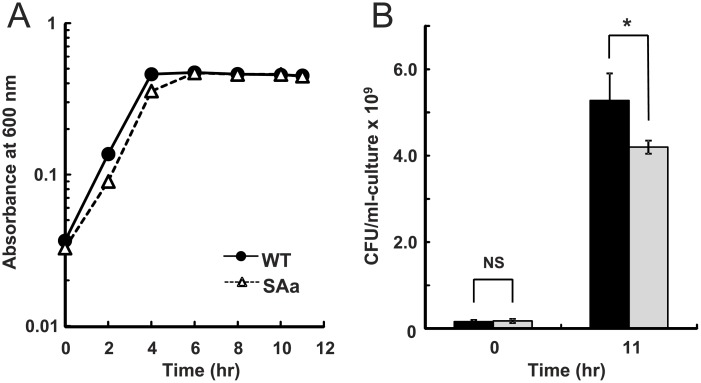
Effects of knockout of the *cox* genes encoding the *caa*_3_ oxidase. **(A)** Growth profiles of the WT (filled circles) and *cox* mutant strain SAa (open triangles) under carbon-limited conditions. The strains were grown aerobically in 3 ml of synthetic medium supplemented with 0.6 g/l glucose in test tubes with shaking at 200 rpm. **(B)** Colony-forming units (CFU) of the strains on LB plates. CFUs were counted immediately after inoculating the cells and after cultivation for 11 h in synthetic medium supplemented with 0.6 g/l glucose. Black and gray bars indicate WT and SAa, respectively. The results represent the means based on three measurements. Error bars indicate standard deviations of the means. Asterisk indicates that the mean values are significantly different by unpaired two-tailed Student’s *t* test (*p*<0.05). NS indicates that the means of the two samples are not significantly different.

A knockout mutant of the *cox* genes (PA0105-0108), designated as SAa, was constructed to investigate the physiological role of *caa*_3_. The aerobic growth profile of SAa did not differ from that of the WT in nutrient-rich LB medium or synthetic medium supplemented with carbon sources, i.e., glucose, glutamate, or succinate (data not shown). The *cox* genes are induced under starvation conditions; therefore, we compared the growth of SAa with that of the WT in synthetic medium containing a restricted concentration (0.6 g/l) of glucose. The growth rate and final optical density of SAa were similar to those of WT (Fig 1A). The maximum specific growth rates (μ_max_) for WT (0.64 ± 0.015 h^−1^) and SAa (0.61 ± 0.021 h^−1^) were not statistically different by *t* test (*p*>0.1). However, the number of SAa cells that could form colonies on LB plate in the stationary phase was significantly lower than that of WT (*p*<0.05) (Fig 1B).

Survival of the *caa*_3_ deficient mutant at stationary phase under carbon starvation was investigated by a comparison of competitive index (CI) and relative fitness (w). Equal amounts of precultures of PAO1ut-Sm and SAa-Tc, which were derivatives of the WT and SAa labeled with Sm and Tc resistance genes, respectively, were mixed and cultivated in synthetic medium supplemented with sufficient (8 g/l) or restricted (0.6 g/l) amounts of glucose. The CI and w values were calculated based on the CFU for each strain before and after incubating for 11 h. The CI and w values for SAa-Tc under carbon-rich conditions were 0.73 ± 0.13 and 0.94 ± 0.03 (n = 3), respectively. By contrast, the values were 0.18 ± 0.08 and 0.60 ± 0.08 (n = 3), respectively, under the carbon starvation conditions, which were significantly lower than those under the carbon-rich conditions (*p*<0.01). These results indicate that *caa*_3_ has an advantageous effect under starvation conditions. Because the ETC terminated by *caa*_3_ has the highest efficiency for creating a proton gradient across the cell membrane among the five terminal oxidases of *P*. *aeruginosa* [14], utilization of *caa*_3_ must provide benefit for energy production from poor carbon sources.

### Isolation of suppressor mutants that utilize *caa*_3_ as the only terminal oxidase

To investigate the enzymatic feature and *in vivo* function of *caa*_3_, a quadruple mutant QXAa was constructed by knocking out the four terminal oxidase gene clusters except for the *cox* genes from PAO1ut in the previous study [14]. Unexpectedly, QXAa was unable to grow in any of the test media under different oxygen concentrations, although it grew anaerobically by denitrification, thereby indicating that *caa*_3_ was either not expressed or potentially inactive in the mutant.

**Fig 2:**
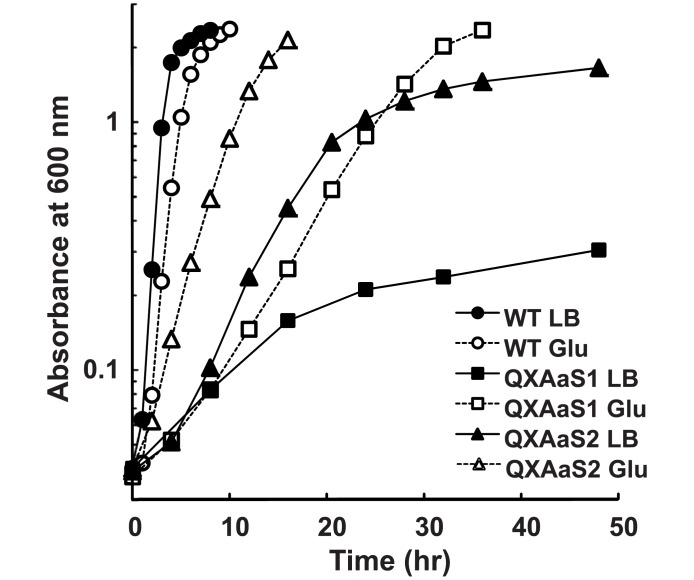
Growth profiles of the suppressor mutants. Strains were cultivated aerobically in 40 ml LB medium or glutamate medium in 100-ml Erlenmeyer flasks with shaking at 300 rpm. The results shown are representative based on three independent experiments. The maximum specific growth rates (μ_max_) of WT, QXAaS1, and QXAaS2 were 1.30 ± 0.089, 0.09 ± 0.008, and 0.26 ± 0.041 h^−1^, respectively, for LB medium and 1.06 ± 0.019, 0.15 ± 0.016, and 0.40 ± 0.034 h^−1^, respectively, for glutamate medium.

The *cox* genes were upregulated in PAO1ut as a starvation response when glutamate was used as the carbon source (data not shown), probably due to the low glutamate uptake activity of the strain [11]. However, QXAa did not grow aerobically in the glutamate medium, suggesting that active terminal oxidase enzyme sufficient to support the aerobic growth was not produced in the mutant. We found that suppressor mutants capable of aerobic growth in the glutamate medium appeared after aerobic incubation for 6–10 days in the medium (S3 Fig). One of the suppressor mutants was isolated from the culture and designated as QXAaS1. The suppressor mutant was not obtained when QXAa was incubated in LB medium for 20 days (S3 Fig). QXAaS1 grew aerobically in the glutamate medium with μ_max_ of 0.15 ± 0.016 h^−1^, but it exhibited poor growth in LB medium with μ_max_ of 0.09± 0.008 h^−1^ (Fig 2). When QXAaS1 was repeatedly subcultured in LB medium under aerobic conditions, second round suppressor mutants capable of faster growth in LB medium appeared in the culture. We isolated two suppressor mutants from different cultures, which we designated as QXAaS2 and QXAaS3. The growth of QXAaS2 was better than that of QXAaS3, thus QXAaS2 was used for further analysis. The growth rate of QXAaS2 was higher than that of QXAaS1 in either LB medium or glutamate medium, but lower than that of WT (*p*<0.01) (Fig 2).

**Fig 3:**
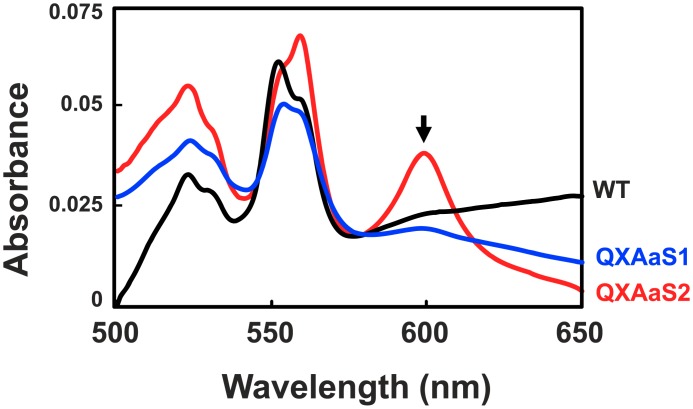
Reduced minus oxidized difference absorbance spectra of membranes from WT, QXAaS1, and QXAaS2. Strains were cultivated aerobically in glutamate medium. Solubilized membrane fractions were oxidized and reduced by ammonium persulfate and sodium dithionite, respectively. The absorption peak at 598 nm specific to heme *a* is indicated by an arrow. Black, blue, and red curves indicate WT, QXAaS1, QXAaS2, respectively.

The expression profiles of cytochromes in the suppressor mutants were investigated by spectral analyses in order to identify semiquantitatively which kind of terminal oxidase was expressed in the mutants. The difference spectra between reduced and oxidized membrane fractions of WT, QXAaS1, and QXAaS2 when grown aerobically in the glutamate medium are shown in Fig 3. The peak at 598 nm corresponding to heme *a* [25, 28] was increased significantly in QXAaS2, but slightly in QXAaS1. Because the *caa*_3_ oxidase has a specific spectral peak of heme *a*, the result suggested that the aerobic growth of the suppressor mutants was supported by the induction of *caa*_3_. The peak was very weak in WT, indicating that the expression level of *caa*_3_ was kept low even when the *cox* genes were induced. The heme *a* contents estimated from the spectral data were 0.08, 0.12, and 1.03 nmol/mg protein for WT, QXAaS1, and QXAaS2, respectively. The peak at 559 nm corresponding to probably heme *b* was also increased in QXAaS2 by unknown reason.

### Identification of the suppressor mutations

**Fig 4:**
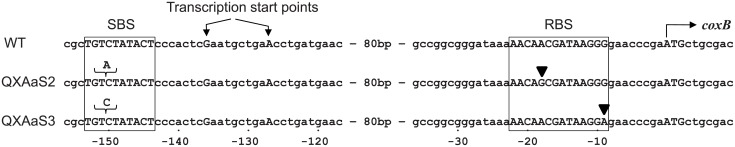
Suppressor mutations in the 5′-flanking region of *coxB* in QXAaS2 and QXAaS3. The putative RpoS binding site (SBS), transcription start points, ribosome binding site (RBS), and the initiation codon of *coxB* are shown in capital letters. Insertion mutations are indicated by braces. Point mutations are indicated by inverted triangles. The numbers below indicate the position relative to the initiation codon of *coxB*.

The change in the expression level of *caa*_3_ in the suppressor mutants was expected to be attributed to the mutations that had occurred in the mutants. To identify the suppressor mutations, the genome of QXAaS2 was sequenced and at least eight loci were found to differ from the genome sequence of PAO1ut (S3 Table). In particular, three mutations were expected to be related to the phenotypes of the suppressor mutants. One was a “C” to “T” substitution at position 85 from the translation initiation codon of *roxS*, which encodes the sensor kinase subunit of RoxSR. This mutation was a nonsense mutation, and thus, QXAaS2 was unable to produce intact RoxSR. RoxSR acts as a repressor of the *coxB* promoter [11], so the transcription of the *cox* genes might be affected by the mutation of *roxS*. The other two mutations were found in the 5′-flanking region of *coxB* (Fig 4). One was an insertion of an “A” at position –151 from the initiation codon of *coxB* and the other was an “A” to “G” point mutation at position –18. No mutations were identified in the protein-coding regions of the *cox* genes. The *coxB* promoter is known to be dependent on a stationary phase sigma factor RpoS [11, 16], but no mutations were identified in *rpoS*.

We also determined the nucleotide sequences of *roxS* and the 5′-flanking region of *coxB* in the genomes of QXAaS1 and QXAaS3. The nonsense mutation in *roxS* was identified in both QXAaS1 and QXAaS3, but QXAaS1 had no mutation in the 5′-flanking region of *coxB*. Two mutations were found in the 5′-flanking region of *coxB* in QXAaS3 (Fig 4). One was an insertion of a “C” at position –151. The insertion position was the same as that in QXSaS2, but the inserted nucleotide was different. The other mutation was a “G” to “A” point mutation at position –9 from the initiation codon of *coxB*. These results indicate that the mutation of *roxS* was necessary for the aerobic growth of QXAa in the glutamate medium and that two additional mutations in the 5′-flanking region of *coxB* were necessary for aerobic growth in LB medium.

QXAa was found to be able to grow aerobically in the glutamate medium when the suppressor mutation disrupted the sensor kinase gene of the two-component regulator RoxSR. This result might suggest that RoxSR repressed the *cox* genes even under the starvation conditions in QXAa. RoxSR is a homolog of RegBA/PrrBA, which senses the redox status or electron flow of the ETC in *Rhodobacter* species [17, 18]. In *P*. *aeruginosa*, RoxSR activates the expression of the *cio* genes encoding CIO [11]. CIO is the only non-heme-copper oxidase in *P*. *aeruginosa* and is highly resistant to respiratory inhibitors, so it should be induced when the other terminal oxidases of the heme-copper oxidoreductase family are inhibited. The *cioA* promoter activity was significantly higher in QXAa than that in the WT or QXAaS1 (*p*<0.01) (S4 Fig). In contrast, the *coxB* promoter was repressed in QXAa even when RpoS was overexpressed. These results indicated that RoxSR was active in QXAa due to the lack of the four terminal oxidases.

### Analysis of the *coxB* promoter

In addition to the mutation of *roxS*, two suppressor mutations in the 5′-flanking region of *coxB* were required for QXAa to grow in the nutrient-rich LB medium. The *coxB* promoter activity was expected to be affected by these mutations. To obtain more information about the *coxB* promoter, the transcription start site was determined by the 5′-RACE analysis using the mRNA purified from the WT cells cultivated in glutamate medium. There were two transcription start points at positions –136 and –127 from the initiation codon of *coxB*, suggesting that *coxB* has two overlapping promoters (Fig 4). Based on the results, the upstream insertion mutations at position –151 were identified as being located near the –10 box and they broke the RpoS binding site (SBS). The downstream point mutations were in the ribosome binding site (RBS) of *coxB*. From these results, we hypothesized that the *coxB* promoter had been made independent of RpoS and that the translation level of *coxB* had been changed by these mutations.

### Effects of the suppressor mutations on the transcriptional activity of the *coxB* promoter

To test these hypotheses, we investigated the effects of the mutations on the transcriptional activity of the *coxB* promoter using *lacZ* transcriptional fusion plasmids. The fragments containing the *coxB* promoter with the WT sequence (wt), the insertion mutation of QXAaS2 at SBS (sbs), the point mutation of QXAaS2 at RBS (rbs), and both mutations of SBS and RBS (sbs + rbs) were inserted upstream of the *lacZ* gene of pQF50 [23], thereby yielding pQF-PcoxB-wt, pQF-PcoxB-sbs, pQF-PcoxB-rbs, and pQF-PcoxB-sbs-rbs, respectively (S2 Fig). The fragment carrying the mutations in QXAaS3 was also used to construct pQF-PcoxB-sbs-rbs (S3). *P*. *aeruginosa* PAO1ut and its *rpoS* mutant RPOS1 and *roxSR* mutant ROX1 were transformed with the constructed *lacZ* fusion plasmids. We determined the transcriptional activities of the fragments containing the *coxB* promoter in the transformants based on the activity of the gene product of *lacZ*, β-galactosidase (Table 1). The cells grown in LB medium were used for the assay.

**Table 1:** Effects of the suppressor mutations on the transcriptional activity of *coxB*.

Strain	Genotype	β-galactosidase activity (Miller unit)				
		wt	sbs	rbs	sbs + rbs	sbs+rbs(S3)
WT	wild-type	39 ± 12	1004 ± 119	35 ± 11	948 ± 87	81 ± 61
RPOS1	Δ*rpoS*	23 ± 6	956 ± 130	20 ± 3	1064 ± 132	93 ± 57
ROX1	Δ*roxSR*	112 ± 21	1251 ± 208	126 ± 6	1228 ± 119	323 ± 125

Strains were transformed with the transcriptional fusion plasmids carrying the 5′-flanking region of *coxB* with the wild-type sequence (wt), the insertion mutation of QXAaS2 at SBS (sbs), the point mutation of QXAaS2 at RBS (rbs), both mutations of QXAaS2 (sbs + rbs), or the mutations of QXAaS3 (sbs + rbs (S3)). The transformed strains were cultivated in 3 ml of LB medium in test tubes with shaking at 300 rpm. β-galactosidase activity was determined when optical density at 600 nm reached 0.35–0.5. Values are means ± standard deviations from three independent cultures.

The activity of the WT promoter was low, but the promoters with the sbs mutation exhibited significantly higher activity than the WT promoter, irrespective of the rbs mutation (*p*<0.01). Significant downregulation in the *rpoS* mutant was not observed for these fragments. These results clearly demonstrate that the *coxB* promoter activity was enhanced by the insertion mutation and it was made independent of RpoS.

The transcriptional activities of the promoter fragments with the sbs mutation did not show significant induction in the *roxSR* mutant. We used a multi-copy vector for the assay, so the copy number of the RoxSR molecules in the cells might have been insufficient for full repression of the promoters on the plasmids.

The transcriptional activities of the fragment with only the rbs mutation showed no significant difference with those of the WT fragment in all strains tested. The activity of the fragment with both the sbs and rbs mutations also showed no significant difference with that of the fragment with only the sbs mutation. These results indicate that the rbs mutation had no effect on the transcriptional activity of the *coxB* promoter.

The fragment with the sbs and rbs mutations from QXAaS3 had a significantly lower transcriptional activity than that from QXAaS2 (*p*<0.01). The activity was slightly higher than that from the WT promoter and the difference was significant only in the *roxSR* mutant (*p*<0.01). Probably, the low transcriptional activity of the fragment from QXAaS3 might have been sufficient to support aerobic growth.

### Effects of the suppressor mutations on the translational activity of *coxB*

To confirm whether the point mutation in RBS affected the translation of *coxB*, *lacZ* translational fusion plasmids were constructed by fusing the initiation ATG triplet of *coxB* in the fragments carrying the promoter with that of the *lacZ* gene from pQF50 (S1 Fig). The growth of the *P*. *aeruginosa* strains transformed with the translational fusion plasmid that carried the mutation fragment of QXAaS2 was significantly inhibited. The cells of the transformant were aggregated in liquid culture, and thus, the β-galactosidase activity could not be determined. The inhibition of growth was probably due to the high level expression of the β-galactosidase protein. Therefore, the mutations in QXAaS3, for which the *coxB* promoter activity was lower than that of QXAaS2 (Table 1), were used in the translational fusion assay.

The upstream region including the initiation ATG triplet of the *lacZ* gene on pQF50 was replaced in-frame with the 5′-flanking region of *coxB* that possessed the WT sequence (wt), the mutation at SBS (sbs), or both mutations at SBS and RBS (sbs + rbs), thereby yielding pQF-PcoxB-wt-TL, pQF-PcoxB-sbs-TL, or pQF-PcoxB-sbs-rbs-TL, respectively (S2 Fig). The *P*. *aeruginosa* strains were transformed with the constructed plasmids and the β-galactosidase activity was determined after cultivation in LB medium (Table 2).

**Table 2:** Effects of the suppressor mutations of the translational activity of *coxB*.

Strain	Genotype	β-galactosidase activity (Miller unit)		
		wt	sbs	sbs + rbs
WT	wild-type	12 ± 8	38 ± 2	641 ± 31
RPOS1	Δ*rpoS*	5 ± 2	40 ± 13	659 ± 53
ROX1	Δ*roxSR*	74 ± 24	242 ± 21	1244 ± 18

Strains were transformed with the translational fusion plasmids carrying the 5′-flanking region of *coxB* with the wild-type sequence (wt), the insertion mutation of QXAaS3 at SBS (sbs), or two mutations of QXAaS3 at SBS and RBS (sbs + rbs). The transformed strains were cultivated in 3 ml of LB medium in test tubes with shaking at 300 rpm. β-galactosidase activity was determined when optical density at 600 nm reached 0.35–0.5. Values are means ± standard deviations from three independent cultures.

The activities of the fragment with the WT sequence were similar to those in the transcriptional assay. In contrast to the results of the transcriptional fusion assay, the activity of the fragment with the sbs mutation was significantly lower than that of the fragment with mutations in both sbs and rbs in any of the strains that we tested (*p*<0.01). These results clearly demonstrate that the rbs mutation contributed to the high level of expression at the translation level.

The *coxBA*-PA0107-*coxC* genes are predicted to be transcribed as a tetracistronic operon [10]. No mutation was found upstream of *coxA*, PA0107, or *coxC*, and thus, the translation levels of these downstream genes might have been coordinated with *coxB* via translational coupling [29].

### Effects of overexpression of the *cox* genes

**Fig 5:**
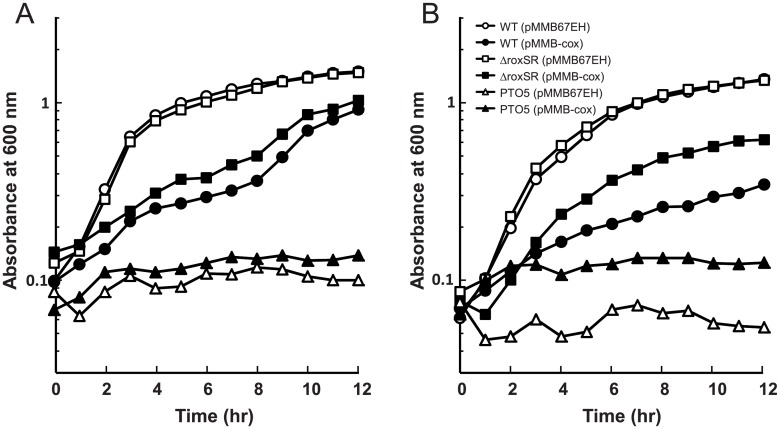
Effect of the overexpression of the *cox* genes. Strains transformed with pMMB67EH or pMMB-cox were cultivated aerobically in 4 ml of LB medium **(A)** and glutamate medium **(B)** in test tubes shaken at 200 rpm. The media were supplemented with 200 μg/ml carbenicillin and 0.5 mM IPTG. The results shown are representative based on at least three independent experiments. The maximum specific growth rates (μ_max_) of WT (pMMB67EH), WT (pMMB-cox), ROX1 (pMMB67EH), and ROX1 (pMMB-cox) were 0.79 ± 0.055, 0.30 ± 0.041, 0.72 ± 0.067, and 0.34 ± 0.103 h^−1^, respectively, for LB medium and 0.65 ± 0.034, 0.27 ± 0.014, 0.67 ± 0.042, and 0.50 ± 0.013 h^−1^, respectively, for glutamate medium. The μ_max_ value was not calculated for the recombinant strains of PTO5, because they did not show apparent growth.

The increased expression level of the *cox* genes appeared to be the primary explanation for the ability of QXAaS2 to grow aerobically. We investigated the effect of overexpression of the *coxBA*-PA0107-*coxC* genes by using a multi-copy expression plasmid. Unexpectedly, when the *cox* genes were overexpressed, a quintuple mutant strain PTO5, which lacked five terminal oxidase gene clusters, was unable to grow aerobically in both LB and glutamate media (Fig 5). The cytochrome *c* oxidase activity was not detected by the Nadi assay by the overexpression of the *cox* genes in the anaerobically grown cells of strain PTO5 (S5 Fig). No activity was also observed when the *cox* genes were overexpressed in the aerobically grown cells of QXCi, which is deficient in the cytochrome *c* oxidase genes but can grow aerobically by using the CIO quinol oxidase [14]. These results indicated that the expression of only the structural genes for the subunits of *caa*_3_ was not sufficient for the function of *caa*_3_. The expression of an active *caa*_3_ requires the production of heme *a* and formation of the heme *a*_3_-Cu_B_ binuclear center. The products of the PA0112–0114 genes, which are located downstream of the *cox* genes, are involved in these processes. The coordinate expression of these accessory proteins and the physiological electron donation system to *caa*_3_ might be necessary for the functional expression of *caa*_3_.

Overexpression of the *cox* genes significantly inhibited the aerobic growth of WT, indicating that the malfunctioning *caa*_3_ has an inhibitory effect on the cell growth (Fig 5). The WT cells were found to produce more ROS by the overexpression of the *cox* genes, suggesting that the growth inhibition was at least partially due to ROS, which was probably produced by the action of the malfunctioning *caa*_3_ (S6 Fig). Cell aggregation was observed in liquid culture when the *cox* genes were overexpressed in the *roxSR* mutant strain ROX1 under aerobic conditions (data not shown), but its aerobic growth was slightly better than that of the WT in LB medium, although the difference in μ_max_ was not significant (*p*>0.1) (Fig 5A). The increase in the ROS generation by the overexpression of the *cox* genes was not observed in the ROX1 cells (S6 Fig). The inhibitory effect of *cox* gene expression in ROX1 was weaker than that in the WT in glutamate medium (Fig 5B). The difference in μ_max_ between the *cox*-overexpression strains of WT and ROX1 was significant (*p*<0.01). RpoS was active in the nutrient poor-glutamate medium, so these results might indicate that the RpoS-dependent gene(s) repressed by RoxSR are required for the expression of a functional *caa*_3_.

The suppressor mutant QXAaS2 exhibited significantly poor growth compared with that of WT (Fig 2). Previously, we reported that the aerobic growth profile of QXCb1, which is a quadruple mutant that only expresses *cbb*_3_-1, was similar to that of WT, although the oxygen consumption activity of the membrane fraction of QXAaS2 was comparable to that of QXCb1 [14]. These results indicate that the poor growth of QXAaS2 was not due to the low cytochrome *c* oxidase activity. The ETC terminated by *caa*_3_ is more efficient at creating a proton gradient across the cell membrane compared with that created by *cbb*_3_-1 [14]. Therefore, the high-level expression of *caa*_3_ might have caused an imbalance in the energy and redox status of the cells. It is also possible that the poor growth of QXAaS2 was due to an inhibitory effect of the malfunctioning *caa*_3_ produced by the increased expression of the *cox* genes.

### Conclusions and future perspectives

In this study, we showed that the expression of *caa*_3_ was advantageous for the survival of *P*. *aeruginosa* under nutrient starvation conditions. However, the expression level of *caa*_3_ was very low even when it was induced and *caa*_3_ was not efficient in supporting cell growth when it was utilized as the only terminal oxidase enzyme. Thus, it is likely that *caa*_3_ functions as a supplementary enzyme to maintain the homeostasis of the redox and energy status in cells via coordination with other terminal oxidases.

The expression of the *cox* genes was found to be tightly regulated by RoxSR and RpoS. These regulatory factors might be also involved in the expression of the putative accessory genes required for the functional expression of *caa*_3_. The translational activity of *coxB* was maintained at low level, even when the *cox* genes were induced. Thus, *caa*_3_ was regulated to avoid its high-level expression at both the transcriptional and translational levels in the WT cells. The overexpression of the *cox* genes also inhibited the aerobic cell growth. The low-level expression of *caa*_3_, and its combination with other terminal oxidases, might be advantageous for the proliferation of *P*. *aeruginosa* in natural oligotrophic environments.

The translated sequence of *coxB* has a heme *c* binding motif in its C-terminus, thus the product of the *cox* gene cluster was predicted to be a *caa*_3_-type enzyme, similar to those found in *T*. *thermophilus* and *Bacillus* species [8, 9]. A γ-proteobacterium *Shewanella oneidensis* MR-1 possesses a *cox* gene cluster similar to that of *P*. *aeruginosa*, but the gene product is probably a *ccaa*_3_-type enzyme because the CoxB subunit has two heme *c* binding motifs in its C-terminal extension [30]. This bacterium also uses the high-affinity *cbb*_3_-type oxidase under aerobic conditions [31]. Recently, Le Las *et al*. reported the expression of a putative *ccaa*_3_-type enzyme under carbon starvation conditions like in the case of *P*. *aeruginosa* [32]. Thus, the utilization of A-type cytochrome *c* oxidases only under starvation conditions might be a common feature of several proteobacteria. Characterizing the enzymatic features of the purified *cox* gene products and identifying the accessory genes required for the functional expression of the active enzyme, as well as the electron donation system, are issues that need to be addressed in future research.

## Supporting information

S1 Fig Schematic representation of the method used for constructing the transcriptional and translational fusion plasmids employed in the *coxB* promoter assay. (PDF)

S2 Fig Schematic representation of the promoter and regulatory regions of the transcriptional and translational fusion plasmids used for the *coxB* promoter assay. (PDF)

S3 Fig Emergence of suppressor mutants that could grow aerobically in glutamate medium under aerobic conditions. The quadruple mutant QXAa and the quintuple mutant PTO5 were cultivated aerobically in 40 ml of LB medium or glutamate medium in 100-ml Erlenmeyer flasks with shaking at 300 rpm.
(PDF)

S4 Fig Effect of the deletion of the terminal oxidase genes on the transcriptional activities of the RoxSR-dependent *coxA* and *cioB* promoters. The bacterial strains that carry the *coxB* or *cioA* promoter fused with *lacZ* were grown anaerobically in LB medium supplemented with 40 mM sodium nitrate. The overnight cultures were diluted ten times in synthetic medium containing no carbon source. The β-galactosidase activities were measured after incubation aerobically for four hours. When necessary, RpoS was overexpressed with pMMBrpoS.
(PDF)

S5 Fig Cytochrome *c* oxidase activity in the transformed strains visualized in blue color by the Nadi assay. (PDF)

S6 Fig ROS generation by overexpression of the *cox* genes. (PDF)

S1 Table Bacterial strains and plasmids used in this study. (PDF)

S2 Table Primers used in this study. (PDF)

S3 Table Mutations identified in QXAaS2. (PDF)
