# Supplementary material for: Specific expression and function of the A-type cytochrome c oxidase under starvation conditions in Pseudomonas aeruginosa
Source: PLoS One. 2017 May 18;12(5):e0177957. doi: 10.1371/journal.pone.0177957 (PMC5436846; doi:10.1371/journal.pone.0177957)
Supplement: S1 Fig — (PDF) [file pone.0177957.s001.pdf]

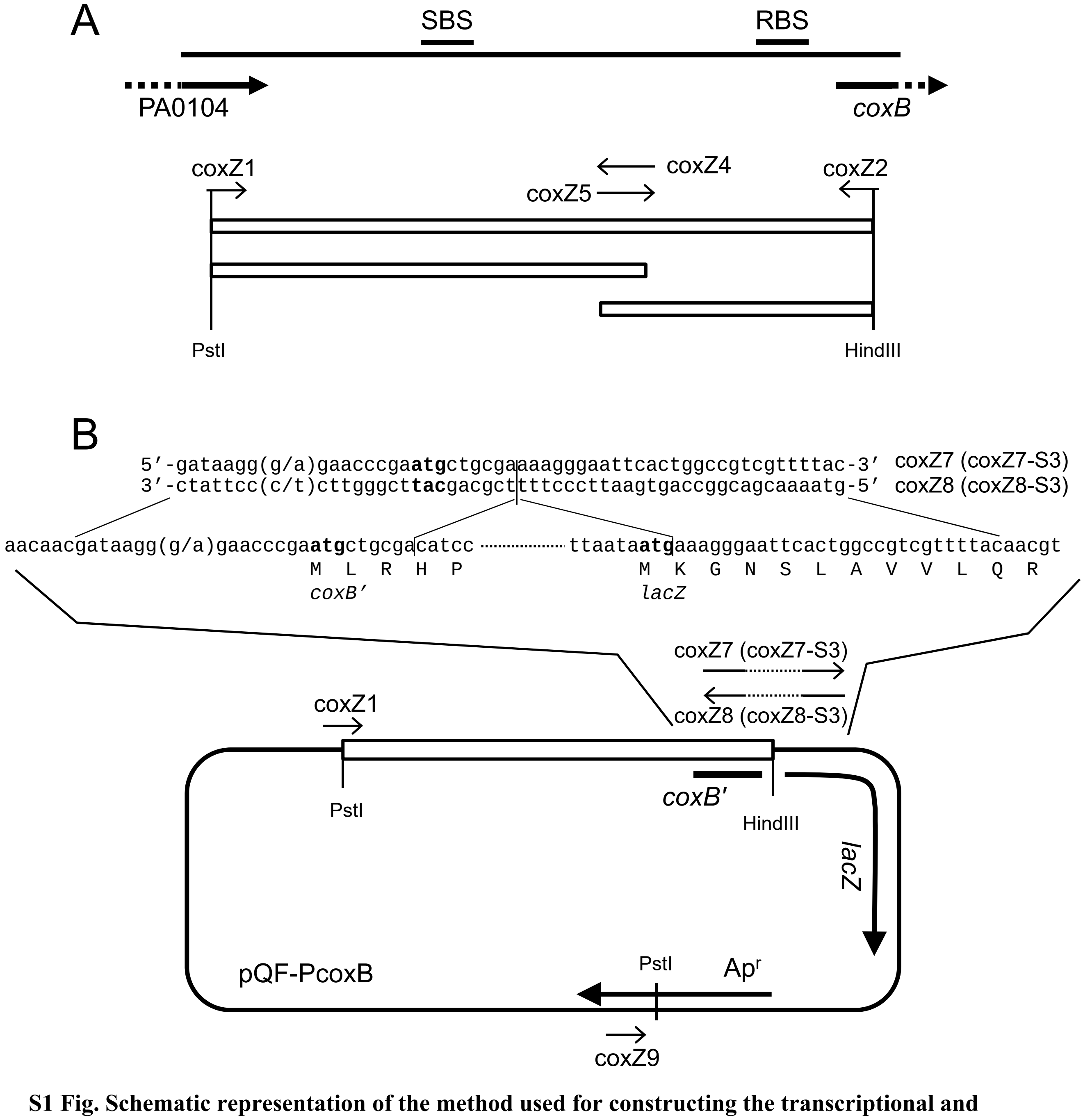

S1 Fig. Schematic representation of the method used for constructing the transcriptional and translational fusion plasmids employed in the *coxB* promoter assay. (A) PCR amplification of the 5′-flanking region of *coxB*. Arrows labeled PA0104 and *coxB* indicate the genes in *P. aeruginosa*. Small arrows indicate the primers used for the PCR reactions. Open bars indicate the amplified PCR fragments. SBS and RBS indicate the RpoS and ribosome binding sites, respectively. (B) Construction of the translational fusion plasmids. pQF-Pcox indicates the transcriptional fusion plasmids that carried the 5′-flanking region of *coxB*. Arrows labeled *lacZ* and Ap^r indicate the β-galactosidase gene and ampicillin resistance gene, respectively. Primers coxZ7 and coxZ8 had the in-frame fusion sequence of *coxB* and *lacZ*. The mixture of PCR fragments amplified from the trancriptional fusion plasmid with the primer sets coxZ1/coxZ8 and coxZ7/coxZ9 was used as the template for the second PCR reaction with primer set coxZ1/coxZ9. The amplified fragment, which carried the translational fusion of *coxB* and *lacZ*, was digested with PstI and ligated with the PstI-digest of pQF50. coxZ7-S3 and coxZ8-S3, which had the point mutation in the ribosome binding site, were used instead of coxZ7 and coxZ8, respectively, to construct the translational fusion plasmids with the mutation.
