# Supplementary material for: Specific expression and function of the A-type cytochrome c oxidase under starvation conditions in Pseudomonas aeruginosa
Source: PLoS One. 2017 May 18;12(5):e0177957. doi: 10.1371/journal.pone.0177957 (PMC5436846; doi:10.1371/journal.pone.0177957)
Supplement: S3 Fig — The quadruple mutant QXAa and the quintuple mutant PTO5 were cultivated aerobically in 40 ml of LB medium or glutamate medium in 100-ml Erlenmeyer flasks with shaking at 300 rpm. (PDF) [file pone.0177957.s003.pdf]

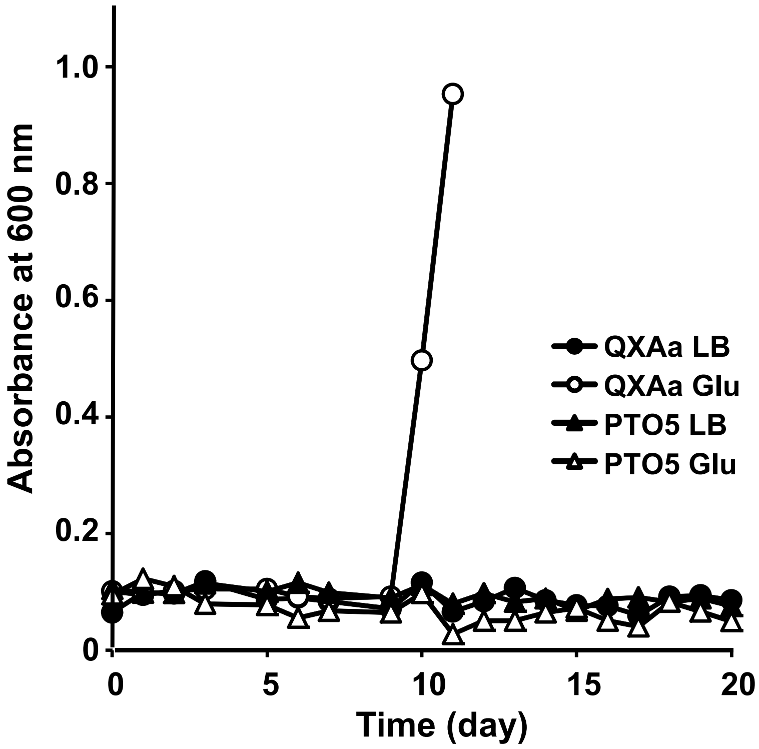

S3 Fig. Emergence of suppressor mutants that could grow aerobically in glutamate medium under aerobic conditions. The quadruple mutant QXAa and the quintuple mutant PTO5 were cultivated aerobically in 40 ml of LB medium or glutamate medium in 100-ml Erlenmeyer flasks with shaking at 300 rpm. The results shown are representative based on more than three independent experiments.
