# Supplementary material for: Specific expression and function of the A-type cytochrome c oxidase under starvation conditions in Pseudomonas aeruginosa
Source: PLoS One. 2017 May 18;12(5):e0177957. doi: 10.1371/journal.pone.0177957 (PMC5436846; doi:10.1371/journal.pone.0177957)
Supplement: S4 Fig — The bacterial strains that carry the coxB or cioA promoter fused with lacZ were grown anaerobically in LB medium supplemented with 40 mM sodium nitrate. The overnight cultures were diluted ten times in synthetic medium containing no carbon source. The β-galactosidase activities were measured after incubation aerobically for four hours. When necessary, RpoS was overexpressed with pMMBrpoS. (PDF) [file pone.0177957.s004.pdf]

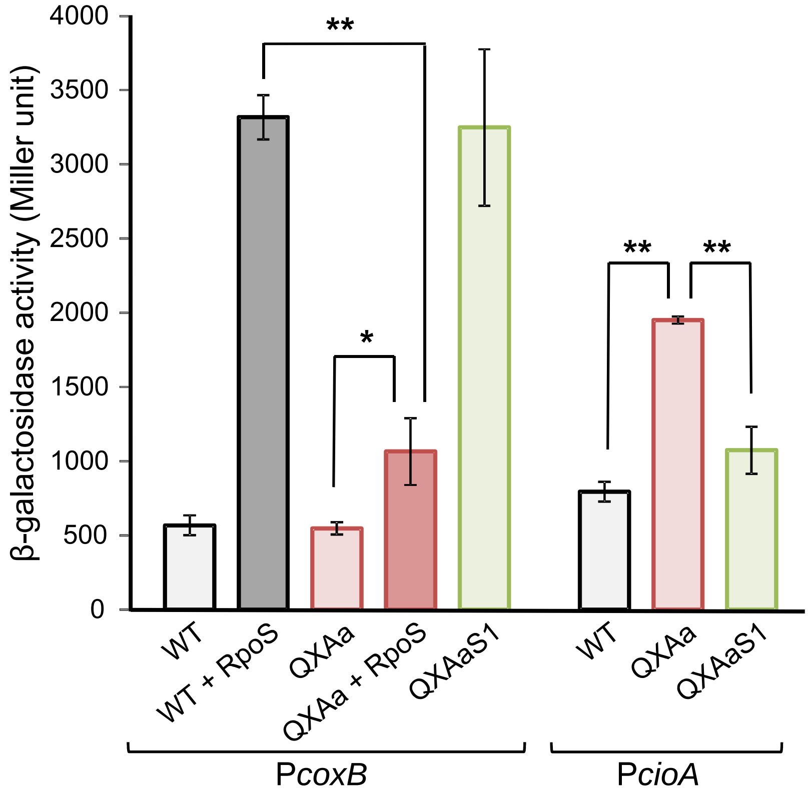

S4 Fig. Effect of the deletion of the terminal oxidase genes on the transcriptional activities of the RoxSR-dependent *coxA* and *cioB* promoters. The bacterial strains that carry the *coxB* or *cioA* promoter fused with *lacZ* were grown anaerobically in LB medium supplemented with 40 mM sodium nitrate. The overnight cultures were diluted ten times in synthetic medium containing no carbon source. The β-galactosidase activities were measured after incubation aerobically for 4 h. When necessary, RpoS was overexpressed with pMMBrpoS. The results represent the means based on at least three independent experiments. Error bars indicate standard deviations of the means. Asterisks indicate that the mean values are significantly different by unpaired two-tailed Student's *t* test (* p<0.05, ** p<0.01).
