# Supplementary material for: Specific expression and function of the A-type cytochrome c oxidase under starvation conditions in Pseudomonas aeruginosa
Source: PLoS One. 2017 May 18;12(5):e0177957. doi: 10.1371/journal.pone.0177957 (PMC5436846; doi:10.1371/journal.pone.0177957)
Supplement: S5 Fig — (PDF) [file pone.0177957.s005.pdf]

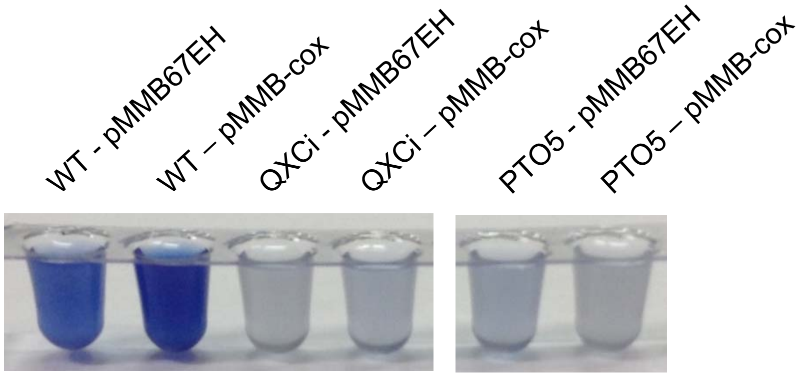

S5 Fig. Cytochrome *c* oxidase activity in the transformed strains visualized in blue color by the Nadi assay. Cells of overnight cultures were suspended in 1 ml of 20 mM Tris-HCl (pH 7.5). After adding 200 µl of a 1:1 mixture of 35 mM α-naphthol in ethanol and 30 mM *N*,*N*-dimethyl-*p*-phenylenediamine monohydrochloride to the cell suspension, the development of blue color by the formation of indophenol blue was observed. The reaction mixtures were incubated for 5 min at room temperature. The picture was taken after the reaction mixtures were moved to a multi-titer plate. The derivatives of PAO1ut (WT) and QXCi were cultivated aerobically in LB medium. QXCi is a quadruple mutant strain that does not have cytochrome *c* oxidases but can grow aerobically by using CIO quinol oxidase. Because PTO5 was unable to grow aerobically, the derivatives of PTO5 were cultivated anaerobically in LB medium supplemented with 40 mM $NaNO_3$. IPTG was added at 0.5 mM to all culture media.
