# Supplementary material for: Specific expression and function of the A-type cytochrome c oxidase under starvation conditions in Pseudomonas aeruginosa
Source: PLoS One. 2017 May 18;12(5):e0177957. doi: 10.1371/journal.pone.0177957 (PMC5436846; doi:10.1371/journal.pone.0177957)
Supplement: S6 Fig — (PDF) [file pone.0177957.s006.pdf]

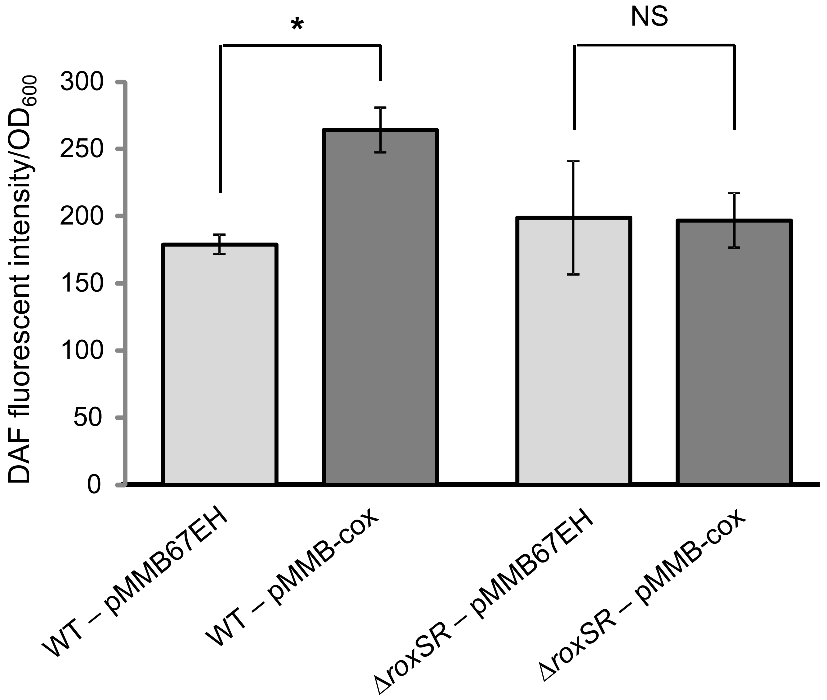

S6 Fig. ROS generation by overexpression of the *cox* genes. Endogenous levels of ROS were assayed with DCFH-DA staining. The bacterial strains were cultivated aerobically in LB medium supplemented with 0.5 mM IPTG. After cultivation for 3 h, a 1 mL aliquot of the culture was mixed with 1 ml of 20 μM DCFH-DA. After incubation for 1 h at 37ºC, cells were collected, washed three times with PBS, and resuspended in PBS. The DAF fluorescence intensities (excitation and emission of 488 and 540 nm, respectively) of cells were measured and normalized by dividing by optical density at 600 nm of the cultures. The results represent the means based on three experiments. Error bars indicate standard deviations of the means. Asterisk indicates that the mean values are significantly different by unpaired two-tailed Student's *t* test ($p<0.01$). NS indicates that the means of the two samples are not significantly different.
