# Supplementary material for: Specific expression and function of the A-type cytochrome c oxidase under starvation conditions in Pseudomonas aeruginosa
Source: PLoS One. 2017 May 18;12(5):e0177957. doi: 10.1371/journal.pone.0177957 (PMC5436846; doi:10.1371/journal.pone.0177957)
Supplement: S1 Table — (PDF) [file pone.0177957.s007.pdf]

S1 Table. Bacterial strains and plasmids used in this study.

Strain or plasmid	Relevant characteristic(s)[a]	Source or reference[b]
P. aeruginosa		
PAO1ut	Our laboratory PAO1 strain used as a wild type	[1]
SAa	Deletion of *coxBA*-PA0107-*coxC*, derivative of PAO1ut	This study
PAO1ut-Sm	PAO1ut labeled with Sm^r	This study
SAa-Tc	SAa labeled with Tc^r	This study
QXAa	Deletion of *cyoABCDE*, *cioAB*, *ccoN1O1Q1P1*, and *ccoN2O2Q2P2*, derivative of PAO1ut	[2]
QXAaS1	Suppressor mutant of QXAa; able to grow aerobically in glutamate medium	This study
QXAaS2	Suppressor mutant of QXAaS1; able to grow aerobically in LB medium	This study
QXAaS3	Suppressor mutant of QXAaS1; able to grow aerobically in LB medium	This study
QXCi	Deletion of *cyoABCDE*, *coxBA*-PA0107-*coxC*, *ccoN1O1Q1P1*, and *ccoN2O2Q2P2*, derivative of PAO1ut	[2]
ROX1	Deletion of *roxSR*, derivative of PAO1ut	[1]
RPOS1	Deletion of *rpoS*, derivative of PAO1ut	[1]
PTO5	Deletion of *cyoABCDE*, *cioAB*, *coxBA*-PA0107-*coxC*, *ccoN1O1Q1P1*, and *ccoN2O2Q2P2*, derivative of PAO1ut	[2]
PAcoxLac	PAO1ut *cox*::*lacZ* transcriptional fusion	[1]
PAcioLac	PAO1ut *cio*::*lacZ* transcriptional fusion	[1]
QXAacoxLac	QXAa *cox*::*lacZ* transcriptional fusion	This study
QXAacioLac	QXAa *cio*::*lacZ* transcriptional fusion	This study
QXAaS1coxLac	QXAaS1 *cox*::*lacZ* transcriptional fusion	This study
QXAaS1cioLac	QXAaS1 *cio*::*lacZ* transcriptional fusion	This study
E. coli		
JM109	Host strain for DNA manipulation	[3]
S17-1	C600::RP-4 2-(Tc::Mu)(Kan::Tn*7*) *thi pro hsdR hsdM$^+$ recA*	[4]
**Plasmids**		
pBR322	Source of Tc^r gene cassette	[3]
pHP45Ω	Source of Sm^r gene cassette	[5]
pGEM-T Easy	Vector for TA cloning, Ap^r	Promega
pMMB67EH	IncQ, expression vector; Ap^r, Cb^r	[6]
pEX-Δcox	Plasmid for *coxBA*-PA0107-*coxC* mutation	[3]
pQF50	*lacZ* promoter probe vector; Ap^r, Cb^r	[7]
pUC18T-mini-Tn*7*T-Gm	Mini-Tn*7*T vector	[8]
mini-Tn*7*-Sm	Derivative of pUC18T-mini-Tn*7*T-Gm used to move Sm^r	This study
mini-Tn*7*-Tc	Derivative of pUC18T-mini-Tn*7*T-Gm used to move Tc^r	This study
pMMB-cox	*coxBA*-PA0107-*coxC* on pMMB67EH	This study
pMMBrpoS	*rpoS* on pMMB67EH	[1]
pQF-PcoxB-wt	*coxB*::*lacZ* transcriptional fusion plasmid with the wild-type promoter sequence, a derivative of pQF50	This study
pQF-PcoxB-sbs	*coxB*::*lacZ* transcriptional fusion plasmid with an insertion mutation of QXAaS2 at the RpoS binding site, a derivative of pQF50	This study
pQF-PcoxB-rbs	*coxB*::*lacZ* transcriptional fusion plasmid with a point mutation of QXAaS2 at the ribosome binding site, a derivative of pQF50	This study
pQF-PcoxB-sbs-rbs	*coxB*::*lacZ* transcriptional fusion plasmid with the mutations of QXAaS2 at the RpoS binding site and ribosome binding site, a derivative of pQF50	This study
pQF-PcoxB-sbs-rbs (S3)	*coxB*::*lacZ* transcriptional fusion plasmid with the mutations of QXAaS3 at the RpoS binding site and ribosome binding site, a derivative of pQF50	This study
pQF-PcoxB-wt-TL	*coxB*::*lacZ* translational fusion plasmid with wild-type promoter sequence, a derivative of pQF50	This study
pQF-PcoxB-sbs-TL	*coxB*::*lacZ* translational fusion plasmid with an insertion mutation of QXAaS3 at the RpoS binding site, a derivative of pQF50	This study
pQF-PcoxB-sbs-rbs-TL	*coxB*::*lacZ* translational fusion plasmid with the mutations of QXAaS3 at the RpoS binding site and ribosome binding site, a derivative of pQF50	This study
pUC-cox-lacZ	Mini-Tn*7*T; *cox*::*lacZ* transcriptional fusion; Ap^r, Gm^r	[1]
pUC-cio-lacZ	Mini-Tn*7*T; *cio*::*lacZ* transcriptional fusion; Ap^r, Gm^r	[1]

a. Ap^r, ampicillin resistant; Cb^r, carbenicillin resistant; Gm^r, gentamicin resistant; Tc^r, tetracycline resistant; Sm^r, streptomycin resistant.
b. The procedures for construction of plasmids and mutant strains are described in the main text.

REFERENCES

1. Kawakami T, Kuroki M, Ishii M, Igarashi Y, Arai H. Differential expression of multiple terminal oxidases for aerobic respiration in *Pseudomonas aeruginosa*. Environ Microbiol. 2010;12(6):1399–412.
2. Arai H, Kawakami T, Osamura T, Hirai T, Sakai Y, Ishii M. Enzymatic characterization and *in vivo* function of five terminal oxidases in *Pseudomonas aeruginosa*. J Bacteriol. 2014;196(24):4206–15.
3. Sambrook J, Fritsch EF, Maniatis T. Molecular cloning: a laboratory manual, 2nd ed. Cold Spring Harbor, NY: Cold Spring Harbor Laboratory Press; 1989.
4. Simon R, Priefer U, Puhler A. A broad host range mobilization system for *in vivo* genetic engineering: Transposon mutagenesis in Gram-negative bacteria. Bio/Technology. 1983;1(9):784–91.
5. Blondelet-Rouault MH, Weiser J, Lebrihi A, Branny P, Pernodet JL. Antibiotic resistance gene cassettes derived from the omega interposon for use in *E. coli* and *Streptomyces*. Gene. 1997;190(2):315–7.
6. Fürste JP, Pansegrau W, Frank R, Blöcker H, Scholz P, Bagdasarian M, *et al.* Molecular cloning of the plasmid RP4 primase region in a multi-host-range *tacP* expression vector. Gene. 1986;48(1):119–31.
7. Farinha MA, Kropinski AM. Construction of broad-host-range plasmid vectors for easy visible selection and analysis of promoters. J Bacteriol. 1990;172(6):3496–9.
8. Choi KH, Schweizer HP. mini-Tn*7* insertion in bacteria with single *att*Tn*7* sites: example *Pseudomonas aeruginosa*. Nat Protoc. 2006;1(1):153–61.
