# Supplementary material for: Specific expression and function of the A-type cytochrome c oxidase under starvation conditions in Pseudomonas aeruginosa
Source: PLoS One. 2017 May 18;12(5):e0177957. doi: 10.1371/journal.pone.0177957 (PMC5436846; doi:10.1371/journal.pone.0177957)
Supplement: S2 Table — (PDF) [file pone.0177957.s008.pdf]

S2 Table. Primers used in this study.

Primer name	Sequence (5' → 3')	Restriction site
pBR-f	cccgaaaagtgccacctgagctctaa	SacII
pBR-r	gacaggagcacgatcatgggtacccg	KpnI
coxB-P	atcatcgaccagaa	
coxB-A1	gcattcgcctggctgatcgc	
coxB-A2	gaagcccatccagactcgtg	
coxB-S1	aggtgagccggtccgtcttc	
coxB-S2	cttctggatctgcgtggtga	
coxZ1	tgagtatgtctgcagtgttctcgccttg	PstI
coxZ2	agcaaaaggaagcttatccagactcgtg	HindIII
coxZ4	ggaccgccaagacaacgaactttgaccgtcgcacctc	
coxZ5	cgaggtgcgacggtcaaagttcgttgtcttggcggtcc	
coxZ7	gataaggggaacccgaatgctgcgaaaagggaattcactggccgtcgttttac	
coxZ8	gtaaaacgacggccagtgaattcccttttcgcagcattcgggttctccttatc	
coxZ9	tccatccagtctattaattgttgccgg	
PAcox_F1	aaggggaattcgaatgctgcgacatcca	EcoRI
PAcox_R1	catttaagcttcccccggtggcggtcag	HindIII
