# Supplementary material for: Specific expression and function of the A-type cytochrome c oxidase under starvation conditions in Pseudomonas aeruginosa
Source: PLoS One. 2017 May 18;12(5):e0177957. doi: 10.1371/journal.pone.0177957 (PMC5436846; doi:10.1371/journal.pone.0177957)
Supplement: S3 Table — (PDF) [file pone.0177957.s009.pdf]

S3 Table. Mutations identified in QXAaS2.

Mutations predicted to be involved in the aerobic growth of QXAaS2 are shown in bold.

Mutation type	Position on the genome of PAO1	Original sequence	Mutated sequence	Description
Substitution	48583	C	T	Silent mutation in PA0041 encoding probable hemagglutinin
**Insertion**	**127227**	**-**	**A**	**Upstream of PA0105 (*coxB*) encoding *aa*$_3$ cytochrome *c* oxidase**
**Substitution**	**127360**	**A**	**G**	**Upstream of PA0105 (*coxB*) encoding *aa*$_3$ cytochrome *c* oxidase**
Substitution	223726	A	G	Upstream of PA0195 (*pntA*) encoding NAD(P) transhydrogenase
Deletion	2700069-2700071	GCG	---	Single amino acid deletion in PA2416 (*treA*) encoding trehalase
Substitution	4894325	G	A	Upstream of PA4367
**Substitution**	**5030164**	**G**	**A**	**Nonsense mutation in PA4494 (*roxS*) encoding sensor kinase of two-component regulator**
Substitution	5744504	G	T	Single amino acid substitution in PA5100 (*hutU*) encoding urocanase
