# Supplementary material for: Specific expression and function of the A-type cytochrome c oxidase under starvation conditions in Pseudomonas aeruginosa
Source: PLoS One. 2017 May 18;12(5):e0177957. doi: 10.1371/journal.pone.0177957 (PMC5436846; doi:10.1371/journal.pone.0177957)
Supplement: S2 Fig — (PDF) [file pone.0177957.s002.pdf]

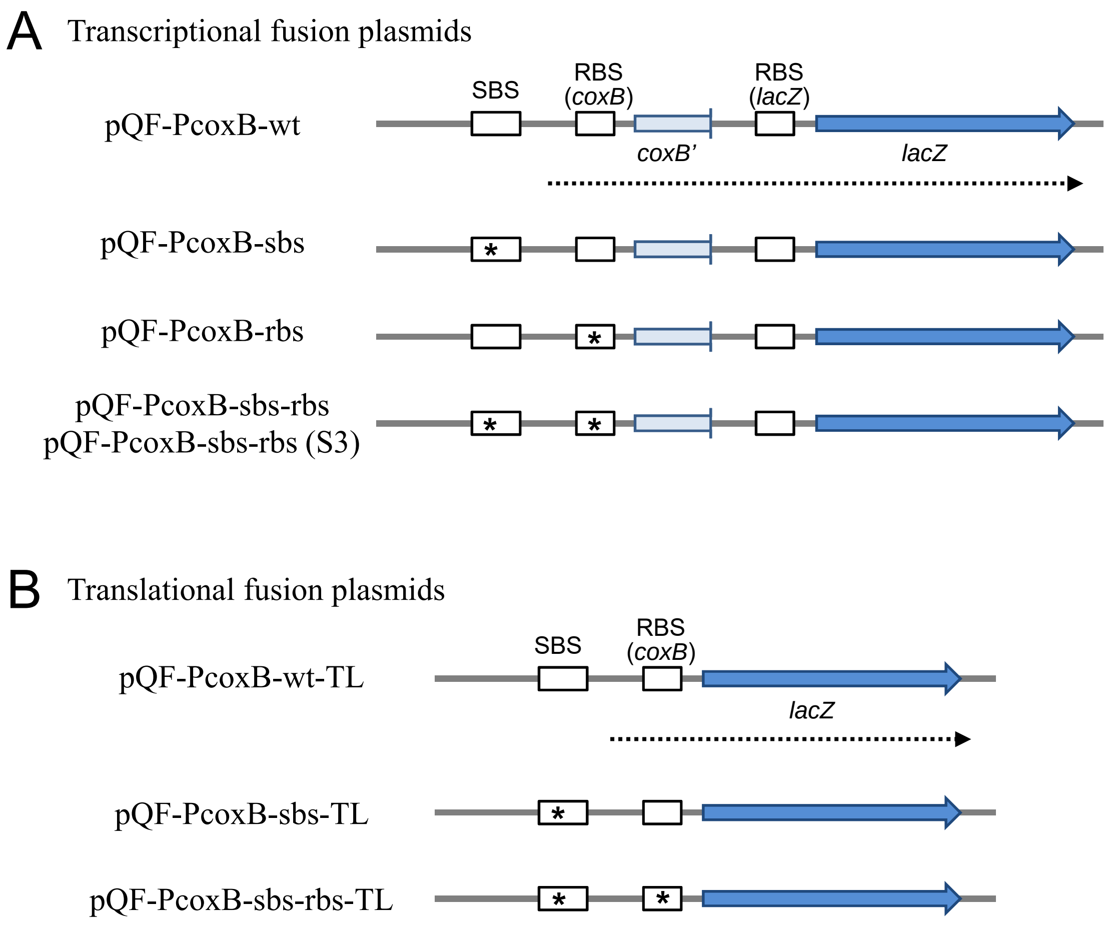

S2 Fig. Schematic representation of the promoter and regulatory regions of the transcriptional and translational fusion plasmids used for the *coxB* promoter assay. (A) Transcriptional fusion plasmids. (B) Translational fusion plasmids. SBS indicates the RpoS binding site. RBS (*coxB*) and RBS (*lacZ*) indicate and ribosome binding sites for *coxB* and *lacZ*, respectively. Asterisks indicate suppressor mutations. Predicted mRNAs were indicated by dashed arrows.
